# Population effect of 10-valent pneumococcal conjugate vaccine on nasopharyngeal carriage of *Streptococcus pneumoniae* and non-typeable *Haemophilus influenzae* in Kilifi, Kenya: findings from cross-sectional carriage studies

**DOI:** 10.1016/S2214-109X(14)70224-4

**Published:** 2014-07

**Authors:** Laura L Hammitt, Donald O Akech, Susan C Morpeth, Angela Karani, Norbert Kihuha, Sammy Nyongesa, Tahreni Bwanaali, Edward Mumbo, Tatu Kamau, Shahnaaz K Sharif, J Anthony G Scott

**Affiliations:** a Kenya Medical Research Institute-Wellcome Trust Research Programme, Kilifi, Kenya; b Department of International Health, Johns Hopkins Bloomberg School of Public Health, Baltimore, MD, USA; c Nuffield Department of Clinical Medicine, University of Oxford, Oxford, UK; d Kenya Ministry of Health, Kilifi, Kenya; e Kenya Ministry of Health, Nairobi, Kenya; f London School of Hygiene & Tropical Medicine, London, UK

## Abstract

**Background:**

The effect of 7-valent pneumococcal conjugate vaccine (PCV) in developed countries was enhanced by indirect protection of unvaccinated individuals, mediated by reduced nasopharyngeal carriage of vaccine-serotype pneumococci. The potential indirect protection of 10-valent PCV (PCV10) in a developing country setting is unknown. We sought to estimate the effectiveness of introduction of PCV10 in Kenya against carriage of vaccine serotypes and its effect on other bacteria.

**Methods:**

PCV10 was introduced into the infant vaccination programme in Kenya in January, 2011, accompanied by a catch-up campaign in Kilifi County for children aged younger than 5 years. We did annual cross-sectional carriage studies among an age-stratified, random population sample in the 2 years before and 2 years after PCV10 introduction. A nasopharyngeal rayon swab specimen was collected from each participant and was processed in accordance with WHO recommendations. Prevalence ratios of carriage before and after introduction of PCV10 were calculated by log-binomial regression.

**Findings:**

About 500 individuals were enrolled each year (total n=2031). Among children younger than 5 years, the baseline (2009–10) carriage prevalence was 34% for vaccine-serotype *Streptococcus pneumoniae*, 41% for non-vaccine-serotype *Streptococcus pneumoniae*, and 54% for non-typeable *Haemophilus influenzae*. After PCV10 introduction (2011–12), these percentages were 13%, 57%, and 40%, respectively. Adjusted prevalence ratios were 0·36 (95% CI 0·26–0·51), 1·37 (1·13–1·65), and 0·62 (0·52–0·75), respectively. Among individuals aged 5 years or older, the adjusted prevalence ratios for vaccine-serotype and non-vaccine-serotype *S pneumoniae* carriage were 0·34 (95% CI 0·18–0·62) and 1·13 (0·92–1·38), respectively. There was no change in prevalence ratio for *Staphylococcus aureus* (adjusted prevalence ratio for those <5 years old 1·02, 95% CI 0·52–1·99, and for those ≥5 years old 0·90, 0·60–1·35).

**Interpretation:**

After programmatic use of PCV10 in Kilifi, carriage of vaccine serotypes was reduced by two-thirds both in children younger than 5 years and in older individuals. These findings suggest that PCV10 introduction in Africa will have substantial indirect effects on invasive pneumococcal disease.

**Funding:**

GAVI Alliance and Wellcome Trust.

## Introduction

Introduction of pneumococcal conjugate vaccine (PCV) into the routine immunisation schedule of developed countries over the past 13 years has resulted in a dramatic reduction in the incidence of invasive pneumococcal disease caused by vaccine serotypes.^1^, ^2^, ^3^, ^4^, ^5^ Additionally, vaccinated individuals are less likely to be carriers of vaccine-serotype pneumococci, and are therefore less likely to transmit the infection, than non-vaccinated individuals. At a population level, vaccination leads to a reduction in the carriage prevalence of vaccine-serotype pneumococci in both vaccinated and unvaccinated individuals and a reduction in the incidence of invasive pneumococcal disease caused by vaccine-serotype pneumococci in the whole population (ie, herd protection). Within 4 years after the introduction of PCV into the childhood immunisation schedule in the USA, the incidence of vaccine-serotype invasive pneumococcal disease in people aged 5 years or older had fallen by 62%.^6^ The indirect protection provided by PCV was greater than its direct protection, and this factor had a profound effect on estimates of the cost-effectiveness of the vaccine.^7^ The nasopharyngeal niche vacated by vaccine-serotype pneumococci is rapidly occupied by non-vaccine-type pneumococci, which has led to serotype replacement disease of varying magnitude in different populations.^5^, ^8^, ^9^, ^10^

Many low-income countries will introduce PCV in the coming decade.^11^ The paucity of robust longitudinal surveillance systems for invasive pneumococcal disease in developing countries poses a challenge in identifying the programmatic effectiveness of PCV in these settings. However, studies of nasopharyngeal carriage, in which the anatomical locus of indirect vaccine effects are investigated, are logistically feasible in developing countries and can help monitor the population effect of PCV.^12^, ^13^, ^14^

In 2011, Kenya became one of the first countries in Africa to introduce PCV and the first country to use the 10-valent PCV conjugated to non-typeable *Haemophilus influenzae* protein-D (PCV10).^15^ Because PCV10 uses protein-D from non-typeable *H influenzae* as its carrier protein, it might induce protection against infections caused by non-typeable *H influenzae*, an important cause of otitis media and respiratory tract infection.^16^ The effect of vaccination on pneumococcal carriage prevalence, and possibly on non-typeable *H influenzae* carriage prevalence, might affect other bacteria in the nasopharynx. An inverse relation between carriage of pneumococcus and *Staphylococcus aureus* has been described, leading to speculation that PCV use might result in an increase in *S aureus* carriage in children, possibly leading to staphylococcal disease.^17^, ^18^, ^19^, ^20^

With support from the GAVI Alliance, PCV10 was introduced into the routine infant vaccination programme in Kenya in January, 2011, with a catch-up campaign for infants. In Kilifi County, a catch-up campaign was also done for all children aged younger than 5 years, which accelerated the population effect of cohort introduction by several years. We aimed to assess the programmatic effects of PCV10 introduction on nasopharyngeal carriage of *Streptococcus pneumoniae*, non-typeable *H influenzae*, and *S aureus* at an early stage.

## Methods

### Study design and participants

The study took place in the Kilifi Health and Demographic Surveillance System (KHDSS), a 891-km^2^ area within Kilifi County, a poor rural district on the Indian Ocean coast of Kenya. The KHDSS has a population of about 260 000 people who have been under surveillance for vital events and migration through 4-monthly household visits since 2000.^21^ *H influenzae* type b conjugate vaccine was introduced into this area in 2001; coverage for three doses of *H influenzae* type b vaccine was 95% at 12 months of age among residents of the KHDSS in 2007.^22^

In January, 2011, the government of Kenya introduced PCV10 into the national immunisation schedule, administered simultaneously with pentavalent vaccine (diphtheria, whole cell pertussis, tetanus, hepatitis B, and *H influenzae* type b combined vaccine) at age 6, 10, and 14 weeks. In 2011, all infants were encouraged to present for a three-dose catch-up schedule 4 weeks apart. In Kilifi County, an additional catch-up campaign was undertaken to provide up to two doses of PCV10 to children aged 12–59 months in two outreach campaigns, beginning on Jan 31, 2011, and March 21, 2011, and lasting 1–2 weeks. These campaigns were managed by the Ministry of Public Health and Sanitation at 45 community health facilities.

We did annual cross-sectional studies of nasopharyngeal carriage in the KHDSS in the 2 years before and 2 years after introduction of PCV10. For each year of the study, we used a Stata program to randomly select 50 residents in each of ten age strata (0, 1–2, 3–4, 5–9, 10–14, 15–19, 20–39, 40–59, 50–59, and ≥60 years) from the KHDSS population register. Using the same method, we randomly selected 30 additional residents in each age strata to serve as a back-up list to cater for people who were lost to follow-up or declined to participate. Participants included in the first year were not excluded from future selection. During June–October, fieldworkers visited the home of each potential participant, explained the study, and obtained written informed consent from each adult participant or from the parent or guardian of each participant aged younger than 18 years. The protocol was approved by the Oxford Tropical Ethical Review Committee (number 30-10) and the Kenya National Ethical Review Committee (SSC1433).

### Procedures

Fieldworkers administered a short questionnaire eliciting risk factors for carriage, documented vaccination history from the immunisation cards of children, and then collected a nasopharyngeal swab specimen. Residents who had moved out of the KHDSS, could not be located, or declined to participate were replaced by choosing the first remaining name from a back-up random selection of residents in each age stratum. A nasopharyngeal rayon swab (Medical Wire, Corsham, UK) specimen was collected from each participant. Specimens were collected by passing the swab through the nostril, along the floor of the nasal cavity until it touched the posterior nasopharyngeal wall, where it was left for 2–3 s, rotated, and removed. Swabs were placed in skim-milk tryptone glucose glycerol media and processed at the Kenya Medical Research Institute-Wellcome Trust Research Programme Laboratory (Kilifi, Kenya), in accordance with WHO recommendations.^23^ Isolates of *S pneumoniae* were identified from gentamicin-blood agar by optochin susceptibility testing; serotyping was done by latex agglutination and the Quellung reaction (including separate antisera for serotypes 6A and 6C). If pneumococcal colonies of varying appearance occurred, only those of the dominant colony morphology were serotyped. All serogroup 6 isolates were retested by PCR for confirmation of serotype. Isolates of *H influenza*e were identified from bacitracin-chocolate agar by gram stain and X and V factor dependence. Typing of *H influenzae* isolates was done by multiplex PCR using an IgA target that discriminates between *H influenzae* and *Haemophilus haemolyticus*, a *bex*A target, to identify encapsulation and specific targets for each capsular type.^24^, ^25^ Isolates of suspected *S aureus* identified from mannitol salt agar were subcultured and identified by gram stain and biochemical testing.

### Statistical analysis

Vaccine serotypes were defined as those contained in PCV10 (1, 4, 5, 6B, 7F, 9V, 14, 18C, 19F, and 23F). Nasopharyngeal carriage prevalence was estimated in four broad age strata for each bacterial group (vaccine-serotype and non-vaccine-serotype pneumococci, non-typeable *H influenzae*, and *S aureus*). Unadjusted prevalence ratios were calculated for the vaccine period (2011–12) compared with the baseline period (2009–10) using classic methods for estimation of risk ratios. To identify potential confounders, we tested the association between all questionnaire variables and the vaccine period. For consistency across models of different age groups and bacterial groups, we adjusted all models for each of the confounding variables that was significant at a p value of less than 0·1. To obtain unconfounded estimates of prevalence ratios in the vaccine period compared with the baseline period, we explored both secular changes in carriage prevalence and secular changes in potential confounders. Prevalence ratios were modelled using log-binomial regression; if the models failed to converge, we used Poisson regression with robust 95% CIs.^26^

Variation in the effect of PCV10 on carriage prevalence with age was tested as an interaction term. Changes in prevalence over time, adjusted for vaccine period, were tested per year of study. Adjusted prevalence ratios were age standardised in ten strata, to represent the stratified sampling scheme, by the inverse of the sampling ratio as population weights; the reference was the KHDSS population register at the midpoint of the study (Jan 1, 2011).

The significance of vaccine effect on carriage of 25 individual serotypes was tested using a Bonferroni correction (ie, 0·05/25). Vaccine effectiveness against carriage (VE_carr_) was calculated as 1 minus the age-standardised, adjusted prevalence ratio. Estimates of vaccine effectiveness against acquisition were calculated as 1 minus the age-standardised, adjusted odds ratio.^27^

All statistical analyses were done using Stata version 11.2.

### Role of the funding source

This work was done under a collaborative arrangement with the PenumoADIP at Johns Hopkins Bloomberg School of Public Health and funded by the GAVI Alliance. This study was done at a research unit funded by the Wellcome Trust of Great Britain. The funders of the study had no role in study design, data collection, data analysis, writing of the report, or the decision to submit manuscript for publication. LLH had full access to all the data in the study, takes responsibility for the integrity of the data and the accuracy of the data analysis, and had final responsibility for the decision to submit for publication.

## Results

Overall, 2031 participants were enrolled (506 in 2009, 511 in 2010, 504 in 2011, and 510 in 2012). The proportion of approached individuals who consented to participate was similar each year (2009: 78%, 2010: 72%, 2011: 71%, 2012: 77%) as were the epidemiological characteristics of participants (table 1). Among participants younger than 1 year, receipt of at least two doses of PCV10 was documented in 38 of 44 (86%; 95% CI 73–95) in 2011 and 43 of 55 (78%; 65–88) in 2012; five (5%) of 99 were completely unvaccinated (n=2) or had unknown vaccination status (n=3). Among participants aged 1–4 years, receipt of at least one dose of PCV10 was documented in 67 of 107 (63%, 95% CI 53–72) in 2011 and in 80 of 109 (73%; 64–81) in 2012. After applying vaccine coverage levels in the study sample to the age distribution of the population, the age-standardised vaccine coverage for receipt of at least one dose of PCV10 among participants younger than 5 years was 69% (95% CI 62–76) in 2011 and 79% (72–86) in 2012. The corresponding figures for all KHDSS residents younger than 5 years were 63% in 2011 and 67% in 2012.^28^

**Table 1:** Epidemiological characteristics of participants

		**2009 (n=506)**	**2010 (n=511)**	**2011 (n=504)**	**2012 (n=510)**
Sex					
	Men	223 (44%)	236 (46%)	241 (48%)	233 (46%)
	Women	283 (56%)	275 (54%)	263 (52%)	277 (54%)
Age					
	<5 years	152 (30%)	156 (31%)	151 (30%)	164 (32%)
	5–17 years	126 (25%)	128 (25%)	125 (25%)	130 (25%)
	18–49 years	124 (25%)	121 (24%)	122 (24%)	116 (23%)
	≥50 years	104 (21%)	106 (21%)	106 (21%)	100 (20%)
Urban residence		60 (12%)	65 (13%)	62 (12%)	72 (14%)
Cough or rhinorrhoea (in preceding 14 days)		257 (51%)	357 (70%)	301 (60%)	298 (58%)
Antibiotic use (in preceding 14 days)		13 (3%)	27 (5%)	39 (8%)	18 (4%)
Smoker in household		116 (23%)	136 (27%)	122 (24%)	102 (20%)
Smoker (if aged ≥18 years)		29/228 (13%)	37/227 (16%)	29/228 (13%)	21/216 (10%)
Daycare attendance (if aged <5 years)		10/152 (7%)	28/156 (18%)	18/151 (12%)	27/164 (16%)
Number of people sharing a bed		1·8 (1–3)	1·4 (1–2)	1·4 (1–2)	1·3 (1–2)
Number of children aged <10 years in household		1·9 (1–3)	2·6 (1–4)	2·3 (1–3)	2·2 (1–3)

Data are number (%) or mean (IQR). Some percentages do not total 100 because of rounding.

A total of 872 pneumococci, 624 *H influenzae*, and 143 staphylococci were isolated. An additional three to five isolates per year were identified as possible pneumococci on the basis of optochin testing but were non-typeable and were excluded from this analysis. Two serotype 6C isolates were detected: one in 2009 that was detected by PCR alone and one in 2012 that was detected by both the Quellung reaction and PCR. Among isolates of *H influenzae*, 588 (94%) were non-typeable *H influenzae* by PCR.

**Figure 1:**
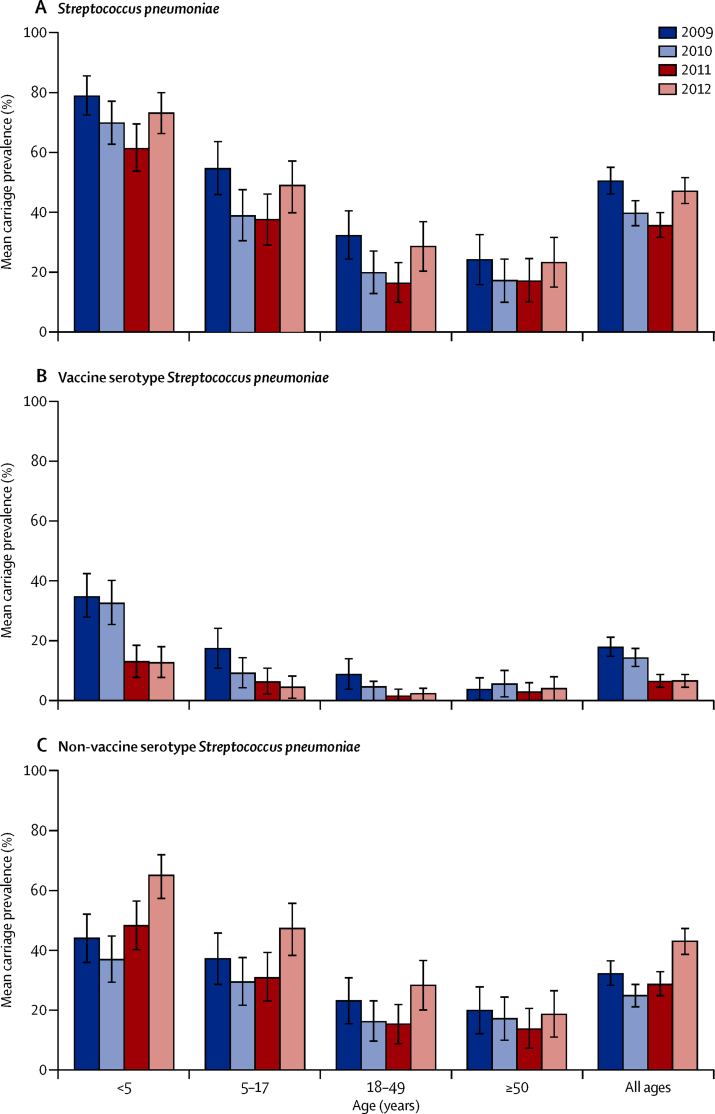
Nasopharyngeal carriage prevalence of *Streptococcus pneumoniae*, vaccine-serotype *S pneumoniae*, and non-vaccine-serotype *S pneumoniae* Bars are 95% CIs.

**Figure 2:**
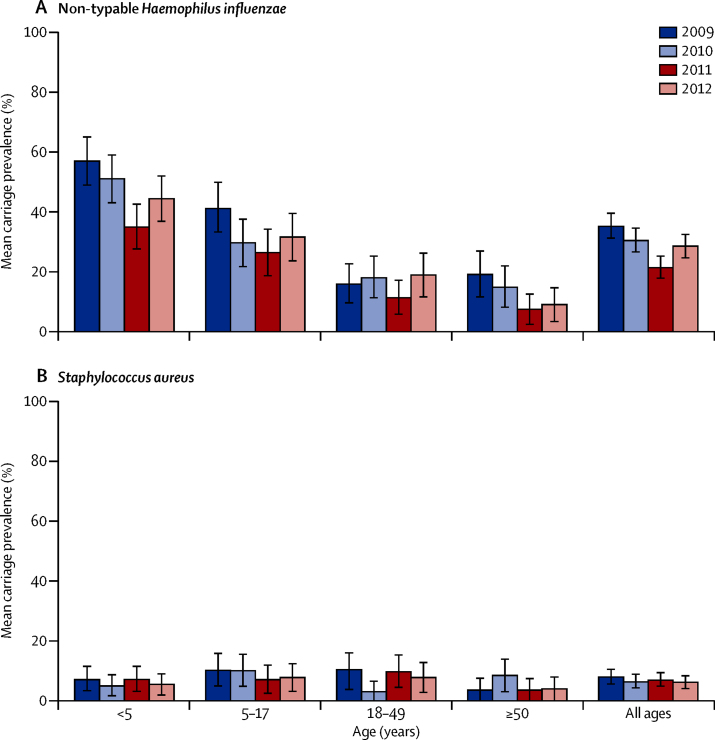
Nasopharyngeal carriage prevalence of non-typeable *Haemophilus influenzae* and *Staphylococcus aureus* Bars are 95% CIs.

Figure 1 shows the pneumococcal carriage prevalence for each of the 4 years of the study among participants in the four age groups. Figure 2 shows results for non-typeable *H influenzae* and *S aureus*. After adjusting for vaccine period and month of sampling within a given year, there were no significant secular changes in carriage prevalence for any of the five bacterial groups tested either in participants younger than 5 years or aged 5 years or older. Among ten epidemiological variables tested for association with vaccine period (table 1), three were significant (p<0·1): month of sampling within a given year (p<0·0001), number of people with whom the participant shared a bed (p=0·0004), and self-reported use of antibiotics within the 14 days before swab collection (p=0·08). All subsequent prevalence ratios were adjusted for these three factors.

Table 2 details the carriage prevalence in the baseline and vaccine periods, crude prevalence ratios, and age-standardised, adjusted prevalence ratios for each of the five bacterial classifications. The adjusted prevalence ratios did not vary significantly by age specified in four strata (<5, 5–17, 18–49, ≥50 years). Consequently, the data are presented for simplicity in two age strata: those targeted for vaccination (age <5 years) and those who were not targeted for vaccination (age ≥5 years). Even in these two strata, the adjusted prevalence ratios did not differ significantly with age for any of the bacterial groups examined.

**Table 2:** Carriage prevalence and prevalence ratios for nasopharyngeal carriage

	**Carriage prevalence baseline period (2009–10)**	**Carriage prevalence vaccine period (2011–12)**	**Crude prevalence ratio (95% CI)**	Age-standardised adjusted prevalence ratio (95% CI)*
**Vaccine-serotype *Streptococcus pneumoniae***				
<5 years	104/308 (34%)	41/315 (13%)	0·39 (0·28–0·53)	0·36 (0·26–0·51)
≥5 years	59/709 (8%)	25/699 (4%)	0·43 (0·27–0·68)	0·34† (0·18–0·62)
**Non-vaccine-serotype *S pneumoniae***				
<5 years	125/308 (41%)	179/315 (57%)	1·40 (1·19–1·65)	1·37 (1·13–1·65)
≥5 years	167/709 (24%)	186/699 (27%)	1·13 (0·94–1·35)	1·13 (0·92–1·38)
**All *S pneumoniae***				
<5 years	229/308 (74%)	213/315 (68%)	0·91 (0·82–1·01)	0·87‡ (0·77–0·97)
≥5 years	226/709 (32%)	204/699 (29%)	0·92 (0·78–1·07)	0·85 (0·71–1·03)
**Non-typeable *Haemophilus influenzae***				
<5 years	167/308 (54%)	126/315 (40%)	0·74 (0·62–0·87)	0·62‡ (0·52–0·75)
≥5 years	168/709 (24%)	127/699 (18%)	0·77 (0·62–0·94)	0·71 (0·56–0·89)
***Staphylococcus aureus***				
<5 years	19/308 (6%)	20/315 (6%)	1·03 (0·56–1·89)	1·02 (0·52–1·99)
≥5 years	56/709 (8%)	48/699 (7%)	0·87 (0·60–1·26)	0·90 (0·60–1·35)

Data are n/N (%).

* Adjusted for month of swab collection, number of people sharing a bed, and antibiotic use in the 14 days preceding swab collection.

† The frequency of vaccine-type pneumococci among participants aged 20–39 years in the vaccine period was zero; this stratum was combined with the group aged 40–49 years for the age–standardised analysis.

‡ Binomial regression model data did not to converge so results of a Poisson model are presented.

For vaccine-serotype *S pneumoniae*, the VE_carr_ was 64% (95% CI 49–74) among children younger than 5 years and 66% (38–82) among individuals aged 5 years or older (table 2). For non-typeable *H influenzae*, the VE_carr_ was 38% (95% CI 25–48) among those younger than 5 years and 29% (11–44) among individuals aged 5 years or older. Among children younger than 5 years, the estimates of VE_carr_ in 2011 and 2012 were 63% (95% CI 44–76) and 62% (38–76), respectively, for vaccine-serotype pneumococci, and 43% (26–56) and 26% (9–40), respectively, for non-typeable *H influenzae*. In an exploratory post-hoc analysis, we compared the VE_carr_ for non-typeable *H influenzae* in 2011 and 2012 among individuals aged 5 years or older and found that the decline was no longer significant 2 years after introduction of PCV10 (2011 VE_carr_ 38%, 95% CI 16 to 54 *vs* 2012 VE_carr_ 23%, −2 to 42). The appendix shows estimates of vaccine effectiveness against acquisition of carriage for each of the five bacterial classifications.

We also examined the effect of vaccination at an individual level in an exploratory post-hoc anaylsis. During the vaccine period, among children 1–4 years old, the adjusted prevalence ratio for those who received at least two doses of PCV10 compared with those who received zero or one doses was 0·47 (95% CI 0·21–1·03) for vaccine-serotype pneumococci and 1·22 (0·87–1·70) for non-typeable *H influenzae*. Because of high vaccine uptake, we were not able to do this analysis among infants, or to compare children who received no doses with those who received at least one dose.

**Figure 3:**
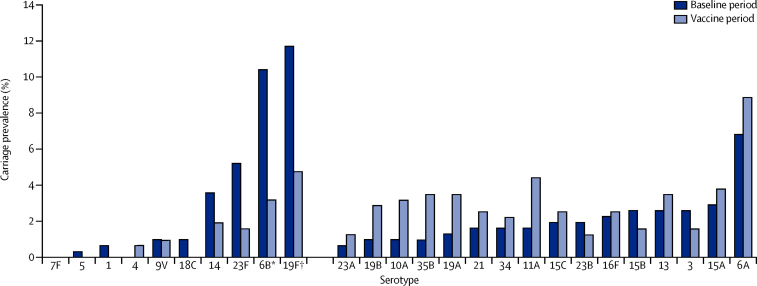
Serotype-specific carriage prevalence of *Streptococcus pneumoniae* among children younger than 5 years *p=0·0003. †p=0·002.

Figure 3 shows the serotype-specific carriage prevalence among children younger than 5 years for the baseline and vaccine periods. The differences in carriage prevalence were significant only for serotypes 6B (10% *vs* 3%; p=0·0003) and 19F (12% *vs* 5%; p=0·002). No effects on carriage of the vaccine-related serotypes 6A or 19A were noted.

## Discussion

We report rapid, significant reductions in vaccine serotype nasopharyngeal carriage at a population level in Kilifi, Kenya, after introduction of PCV10 into the routine infant vaccination schedule accompanied by a catch-up campaign for children younger than 5 years. To our knowledge, this is the first study to report the effects on carriage of a national PCV vaccination programme in a GAVI Alliance-eligible developing country (panel). Although pneumococcal carriage is often asymptomatic and benign, it is a necessary precursor in the development of invasive disease. Because of this causal link, vaccine effect on carriage is an important marker of vaccine-induced protection against disease in children and adults.^13^

Panel **Research in context**

**Systematic review**

After trials of pneumococcal conjugate vaccine (PCV) in the USA, The Gambia, and South Africa showed an excellent vaccine efficacy against vaccine-serotype invasive pneumococcal disease,^29^, ^30^, ^31^, ^32^ WHO recommended that PCV should be included in the routine immunisation schedules of developing countries and several funding agencies pledged support for this introduction. Kenya was chosen as one of the first countries in Africa to receive support for vaccine introduction from the GAVI Alliance. The Kenya PCV Impact Study^28^ was designed to assess the effectiveness and cost-effectiveness of PCV in a setting where it was possible to investigate the effect of PCV against a background of longitudinal surveillance. Several studies in developed countries have established a strong association between vaccine effect on carriage and vaccine effect on invasive pneumococcal disease.^33^, ^34^, ^35^, ^36^, ^37^, ^38^ The nasopharyngeal carriage study of the Kenya PCV Impact Study was designed to provide an early assessment of vaccine effect in a developing country setting. A formal systematic review was not done as part of the study.

**Interpretation**

In this study, introduction of PCV10 in a developing country setting, with a catch-up campaign, led to a two-thirds reduction in carriage prevalence of vaccine-serotype pneumococci both in children targeted for vaccination and in older people who were not vaccinated. The effect reported in children provides convincing functional evidence that the vaccine is inducing immunological protection at a level sufficient to prevent invasive disease. The effect in older children and adults suggest that the childhood PCV10 programme is reducing transmission of vaccine-serotype pneumococci within the population and this is likely to lead to a reduction in vaccine-serotype invasive pneumococcal disease across all age groups (ie, herd protection).

About 18 months after the introduction of PCV10, we noted a 64% reduction in vaccine-serotype *S pneumoniae* carriage among children younger than 5 years, 79% of whom had received at least one dose of PCV10. In comparison, in Alaska, USA, 3 years after introduction of 7-valent PCV (PCV7), a 91% reduction in vaccine serotype carriage was noted among Alaska Native children aged 5 years or younger, more than 99% of whom had received at least one dose of PCV7.^33^ In both settings, PCV was introduced with a catch-up campaign. In a large cluster-randomised study in The Gambia,^39^ in which widespread PCV7 vaccination was undertaken, there was a 56% reduction in vaccine serotype carriage in children aged 2–5 years living in villages where children younger than 30 months were vaccinated and a 74% reduction in villages where all residents receive at least one dose of PCV7. Within 2 years after PCV7 was introduced into the public immunisation programme for infants without a catch-up campaign in a South African community with high HIV prevalence, vaccine serotype carriage was reduced by 50% among children younger than 2 years, 51% of whom had received three doses of PCV7.^40^ In the Netherlands, 3 years after introduction of PCV7, without a catch-up campaign, vaccine serotype carriage was reduced by 80–90% among vaccinated children aged between 11 months and 24 months.^34^ In Portugal, 4 years after PCV7 became available, in children aged 4 months to 6 years, 57% of whom had received PCV7, vaccine serotype carriage was reduced by 78%.^35^ The above-mentioned studies show a substantial effect on vaccine serotype carriage 1·5–4 years after introduction of PCV with and without a catch-up campaign, with vaccine coverage in the target age group ranging between 50% and 100%. Reductions in carriage have been matched by reductions in invasive pneumococcal disease in settings where such data are available, including the KHDSS, where 1 year after PCV10 introduction, the vaccine effectiveness was estimated to be 72% (95% CI 34–88) against vaccine-serotype invasive pneumococcal disease in children aged younger than 5 years.^36^, ^37^, ^38^, ^41^

In addition to the effect in the vaccine target age group, we noted a 66% reduction in vaccine serotype carriage adjusted prevalence in individuals aged 5 years or older. This finding is consistent with findings among Alaska Native people, in whom a 68% reduction in vaccine serotype carriage was reported among people aged at least 18 years about 3 years after introduction of PCV7.^33^ The reductions in vaccine serotype carriage in the non-target age group in Kilifi were apparent in the first post-PCV10 survey (2011), when coverage with at least one dose of PCV10 among children younger than 5 years was 63% in the KHDSS and 69% among study participants. Reasons for the difference in the vaccine coverage estimates for study participants compared with all KHDSS children are as follows: (1) the time lag between the random selection of potential participants and their enrolment in the study caused our sample of the very youngest age group (0–12 months) to be skewed towards the upper end of this bracket when children were more likely to have been vaccinated; (2) our study captured vaccinations given outside the area covered by the vaccine registry; and (3) some of the migrant population—who generally have lower levels of vaccine coverage—would have been lost after random selection in our study. Nonetheless, our findings suggest that substantial indirect effects occur when two-thirds of children younger than 5 years are vaccinated and imply that the indirect protection against invasive pneumococcal disease noted in the USA and UK can probably be replicated in developing countries.

In other settings, the reduction in vaccine serotype carriage prevalence after programmatic introduction of PCVs has been matched by a reciprocal increase in carriage prevalence of non-vaccine serotypes (ie, serotype replacement carriage) such that the overall pneumococcal carriage prevalence, typically, is unchanged from baseline. However, serotype replacement in the nasopharynx has had a variable effect on invasive pneumococcal disease.^5^, ^10^ In most settings, serotype replacement invasive pneumococcal disease has been minimal, whereas in some settings it has almost negated the beneficial effect of PCVs in some subgroups of the population. We noted a significant increase in carriage of non-vaccine-serotype pneumococci among children younger than 5 years; however, because the magnitude of the decline in vaccine serotype carriage was greater, there was a slight decline in overall *S pneumoniae* carriage prevalence in the PCV10 period. The reduction in overall pneumococcal carriage in children is likely to be attributable to the vaccine itself, rather than to underlying variations in carriage, because analyses of the change in carriage prevalence for all pneumococci over the 4 years did not identify a significant decline in prevalence after adjusting for vaccine effect. Although non-vaccine serotype carriage increased significantly in children younger than 5 years, the increase was not statistically significant in people aged 5 years or older. Children are likely to experience more rapid, direct clearance of vaccine serotype carriage and subsequent replacement carriage, whereas adults experience delayed, indirect clearance. Thus, replacement carriage in adults is probably delayed. Two other studies in Africa—a cluster-randomised trial of PCV in The Gambia^39^ and an observational ecological study after programmatic introduction of PCV7 in South Africa^40^—found that non-vaccine serotype carriage declined in adolescents and adults after PCV use in children. However, these findings are subject to several limitations including the short period of follow-up and changes in HIV treatment regimens in South Africa, and an intercurrent community-wide azithromycin campaign in The Gambia. Long-term surveillance is essential to understand PCV-induced changes in non-vaccine serotype carriage and disease.

In serotype-specific analyses, we noted no effect on carriage of the serotypes 6A or 19A in the target age group. This finding is consistent with data from clinical trials that show that PCV10 does not induce a robust antibody response against these strains.^42^ Only the predominant colony appearance of pneumococcus was serotyped from each nasopharyngeal swab, so serotype-specific variations do not account for changes that might have occurred among the non-dominant strains carried by an individual. However, assuming that the probability of sampling a strain is proportional to the frequency of that serotype in the nasopharynx then the present study is of a random sample of strains in a random sample of individuals and this limitation should not affect our conclusions about vaccine effectiveness. Carriage is expected to be in flux in the first few years of vaccine use and carriage prevalence will probably continue to change before reaching equilibrium.^43^, ^44^

We noted a significant reduction in nasopharyngeal carriage of non-typeable *H influenzae* among participants younger 5 years and at least 5 years old in the vaccine period compared with baseline. However, the role of PCV10 as the causative agent of this change is questionable since non-typeable *H influenzae* carriage prevalence seemed to rebound in year 2 of the vaccine period and we did not find an association between an individual's vaccination status and carriage of non-typeable *H influenzae* (by comparing individuals with at least two doses to those with zero or one dose). Findings from early clinical trials suggested that use of an 11-valent protein-D conjugate vaccine reduced the carriage prevalence of vaccine-serotype and non-typeable *H influenzae*, although the decline in non-typeable *H influenzae* carriage was not significant when molecular methods were used to differentiate non-typeable *H influenzae* from the closely-related *H haemolyticus*.^16^, ^45^ In long-term follow-up, lower non-typeable *H influenzae* carriage prevalence in vaccine recipients compared with controls was documented at about 2 years of age but at no other timepoint.^46^ Other clinical trials of PCV10 have not documented a significant, consistent effect of vaccination on carriage of non-typeable *H influenzae*.^47^, ^48^, ^49^ Although the prevalence of non-typeable *H influenzae* might have been higher had we collected an oropharyngeal swab in addition to a nasopharyngeal swab, our methods were similar across years of the study, thus allowing comparison between periods before and after vaccination.^50^

We reported no change in the carriage prevalence of *S aureus* after introduction of PCV10. By contrast, findings from several studies have suggested an inverse relation between carriage of *S pneumoniae* and *S aureus,*^17^, ^18^, ^19^, ^20^ and one population-level assessment in the Netherlands reported an increase in *S aureus* carriage after introduction of PCV7.^51^ Potential explanations for this difference include variations in the nasopharyngeal microbiome across populations and the competition dynamics that ensue after reductions in pneumococcal carriage in a rural developing country setting. *S aureus* was cultured in our study, and in most of the comparator studies cited earlier, from the posterior nasopharynx. Cultures of the anterior nares might be more appropriate to fully characterise the effect of the vaccine on *S aureus.* The period after vaccine surveillance in the present study is brief and the sustainability of effects (or absence of effects) on carriage of various different bacteria can only be identified after a longer period of surveillance. We intend to extend surveillance for at least 3 more years, but have reported early results because the catch-up campaign provided additional maturity to the programme and the vaccine effects are large.

In presenting measures of VE_carr_, we used the term effectiveness to describe the magnitude of the effect of the vaccine in the total population (that we sampled randomly) under the short-term conditions of rapid introduction with high coverage. The effect of the programme will evolve over time and will be determined by both the coverage in infants and the age structure of coverage among the total carrier population. In presenting the prevalence ratio, we have assumed that the key risk for disease is total carriage prevalence. If the key risk is acquisition of carriage, then the odds ratio might provide a more accurate estimate. However, the two methods (prevalence ratio and odds ratio) yielded similar results in this analysis.

PCVs are being introduced rapidly across developing countries, although there is, as yet, limited evidence of their operational effect. PCV10, in particular, has not been studied in any national vaccination programme. This study has shown that introduction of PCV10 in a developing country setting, with a catch-up campaign, has led to a two-thirds reduction in carriage prevalence of vaccine-serotype pneumococci both in children targeted for vaccination and in older people who were not vaccinated. The effect reported in children provides convincing functional evidence that the vaccine is inducing immunological protection at a level sufficient to prevent invasive disease. The effect in older children and adults suggests that the childhood PCV10 programme is reducing transmission of vaccine-serotype pneumococci within the population and this is likely to lead to a reduction in vaccine-serotype invasive pneumococcal disease across all age groups (ie, herd protection).

## Supplementary Material

Supplementary appendix

Supplementary audio Laura Hammitt discusses the effectiveness of 10-valent pneumococcal vaccine in Kenya.
