# Supplementary material for: Population effect of 10-valent pneumococcal conjugate vaccine on nasopharyngeal carriage of Streptococcus pneumoniae and non-typeable Haemophilus influenzae in Kilifi, Kenya: findings from cross-sectional carriage studies
Source: Lancet Glob Health. 2014 Jul;2(7):e397–405. doi: 10.1016/S2214-109X(14)70224-4 (PMC5628631; doi:10.1016/S2214-109X(14)70224-4)
Supplement: Supplementary appendix [file mmc1.pdf]

THE LANCET Global Health

Supplementary appendix

This appendix formed part of the original submission and has been peer reviewed. We post it as supplied by the authors.

Supplement to: Hammitt LL, Akech DO, Morpeth SC, et al. Population effect of 10-valent pneumococcal conjugate vaccine on nasopharyngeal carriage of *Streptococcus pneumoniae* and non-typeable *Haemophilus influenzae* in Kilifi, Kenya: findings from cross-sectional carriage studies. *Lancet Glob Health* 2014; published online May 28. http://dx.doi.org/10.1016/S2214-109X(14)70224-4.

eTable 1. Carriage prevalence and odds ratio for nasopharyngeal carriage of *S. pneumoniae,* non-typeable *H. influenzae* and *S. aureus* in participants aged <5 years and ≥5 years, Kilifi, Kenya, 2009 – 2012

	**Carriage Prevalence Baseline period***		**Carriage Prevalence Vaccine Period†**		**Crude Odds Ratio (OR)**		**Age-standardized Adjusted Odds Ratio (aOR)‡**	
	**n**	**%**	**n**	**%**	**OR**	**95% CIs**	**aOR**	**95% CIs**
Vaccine-type *S. pneumoniae*								
<5 years	104	34	41	13	0·29	0·20, 0·44	0·26	0·17, 0·41
≥5 years	59	8	25	4	0·41	0·25, 0·66	0·32§	0·17, 0·60
Non vaccine-type *S. pneumoniae*								
<5 years	125	41	179	57	1·93	1·40, 2·65	1·82	1·27, 2·60
≥5 years	167	24	186	27	1·18	0·92, 1·50	1·17	0·87, 1·57
All *S. pneumoniae*								
<5 years	229	74	213	68	0·72	0·51, 1·02	0·59	0·40, 0·88
≥5 years	226	32	204	29	0·88	0·70, 1·11	0·77	0·58, 1·02
Non-typeable *H. influenzae*								
<5 years	167	54	126	40	0·56	0·41, 0·77	0·40	0·27, 0·58
≥5 years	168	24	127	18	0·71	0·55, 0·93	0·62	0·45, 0·85
S. aureus								
<5 years	19	6	20	6	1·03	0·54, 1·95	1·01	0·50, 2·07
≥5 years	56	8	48	7	0·86	0·58, 1·28	0·89	0·57, 1·39

The model assumes stationarity and that duration of carriage is unchanged by vaccination.[25]
CI = Confidence Interval
*The total sample in 2009-10 was 308 and 709 in <5 and ≥5 year olds, respectively.
†The total sample in 2011-12 was 315 and 699 in <5 and ≥5 year olds, respectively.
‡Adjusted for month of swab collection, number of people sharing a bed and antibiotic use in the 14 days preceding swab collection.
§The frequency of vaccine-type pneumococci among participants aged 20-39 in the vaccine period was zero; this stratum was combined with the group aged 40-49 years for the age-standardized analysis.
